# Adaptation of grassweeds to spring cropping through changes in germination, flowering time and fecundity

**DOI:** 10.1038/s41598-025-04664-3

**Published:** 2025-07-01

**Authors:** Jasper Kanomanyanga, John Cussans, Stephen Moss, Erick Ober, Chun Liu, Shaun Coutts

**Affiliations:** 1 Lincoln Institute for Agri-Food Technology, University of Lincoln, Lincoln, UK; 2 Niab, Cambridge, UK; 3 ADAS Boxworth, Cambridge, UK; 4 Stephen Moss Consulting, Harpenden, Hertfordshire, UK; 5 Syngenta, Jealott’s Hill International Research Centre, Bracknell, Berkshire, UK

**Keywords:** Blackgrass (*Alopecurus myosuroides*), Italian ryegrass (*Lolium multiflorum*), Sowing time, Spring cropping, Plant phenology, Sustainable weed management, Agroecology, Plant evolution, Evolutionary developmental biology, Environmental sciences

## Abstract

The ability of weed populations to adapt is pivotal for their success in overcoming adverse conditions, including control measures and climate change. Here, we show evidence for the local adaptation of blackgrass (*Alopecurus myosuroides*) and Italian ryegrass (*Lolium multiflorum*) to the distinct seasonal windows for crop establishment. We assessed germination, flowering, and fecundity traits in 16 parental populations (eight each of *A. myosuroides* and *L. multiflorum*) sourced from fields with repeated autumn- or spring-cropping backgrounds. We find evidence of contrasting maternal and genetic influences on the response of *A. myosuroides* germination to light exposure. *Alopecurus myosuroides* seeds collected from fields with a spring-cropping background demonstrated higher germination rates (86%) in darkness than seeds collected from fields with an autumn-cropping system (46%), evidence of maternal effects. Conversely, when parent plants from those same autumn and spring background populations were grown under identical conditions, the offspring seeds (one generation removed from the field) from an autumn-cropping linage germinated more in darkness (81%) than those from a spring-cropping linage (42%), evidence of a contrasting genetic effect. Parental biotypes of both species from fields with a spring-cropping history flowered earlier under spring-like vernalisation treatments, including no vernalisation, compared to their autumn counterparts. Additionally, parental seeds from spring-cropping fields of both species had higher seed production under treatments reflecting the cropping history of their parent populations. These findings provide phenotypic evidence for the potential adaptation of *A. myosuroides* and *L. multiflorum* to spring cropping systems. Diversifying cropping practices, such as integrating seasonal rotations within crop establishment windows, is therefore essential for disrupting weed adaptation and promoting sustainable weed management strategies.

**Supplementary Information:**

The online version contains supplementary material available at 10.1038/s41598-025-04664-3.

## Introduction

Weeds are a threat to global crop production, causing substantial yield losses^1^. They exhibit rapid adaptability to a wide range of environments and management practices, including sowing dates^2–4^, complicating their management^5,6^. A current pressing example is herbicide resistance in economically important species such as blackgrass (*Alopecurus myosuroides*)^7–9^ and Italian ryegrass (*Lolium multiflorum*)^10,11^. These species are particularly problematic in European arable systems, where they are predominantly associated with autumn-sown crops. Both have shown remarkable adaptability, including shifts in phenology in response to changing agronomic practices^12–14^. To address the growing control challenges, particularly due to evolved resistance mechanisms, spring crops have been increasingly incorporated into rotations^15^. However, in fields with repeated spring cropping, these weeds may adapt, developing ‘spring-emerging’ traits better suited to the new growing conditions.

Germination timing is pivotal in weed survival, allowing seedlings to emerge under favourable conditions while avoiding pre-sowing weed control practices^16,17^. In arable cropping systems, most weeds are annuals. Thus, the establishment and persistence of populations rely on the germination of a seed reserve that exists within or on the soil surface^18^. For species like *A. myosuroides* and *L. multiflorum*, germination is triggered by suitable conditions, including soil moisture, temperature, and disturbance^19,20^. Most seeds of these species exhibit a relatively short period of innate dormancy, and consequently, most plants emerge in autumn from seeds produced in summer of the same year. However, a smaller proportion have greater dormancy, resulting in some plants not emerging until the next spring^19,21^. This staggered germination may reflect a bet-hedging strategy, wherein maternal plants produce offspring with varied germination to spread risk across unpredictable environments, including cropping systems^22^. In addition to genetic variation, maternal environmental effects can also influence seed dormancy and germination behaviour. In *A. myosuroides*, soil water potential and temperature during reproductive growth control seed dormancy^23^, while conditions during seed production and storage affect germination rates and early seedling development^24^. Farming practices, such as delayed sowing and reduced seed burial in no-till systems, can influence the proportion of seeds germinating in autumn versus spring, disrupting germination and seedling survival during crop establishment^25,26^. For instance, delayed sowing disrupts the germination of autumn-emerging weeds through pre-sowing cultivations, while spring drilling in warmer soils favours seeds with lower dormancy, potentially shifting competitive dynamics between weeds and crops^27,28^. Over time, repeated spring cropping may create selection pressures favouring populations with traits adapted to spring conditions, such as increased dormancy or modified germination timing^27,29^.

The adaptive potential of weed seeds in response to management actions complicates designing effective control strategies. Germination, flowering, and fecundity traits are fundamental for determining the species’ fitness, with the latter strongly linked to the evolution of other traits. These traits interact dynamically with management practices, creating selection pressures that favour traits aligned with specific cropping systems^27^. For example, delayed sowing in spring-drilled systems can select for rapid germination and early flowering weeds, while no-till systems alter seed burial depth and germination cues, favouring seeds adapted to surface conditions^30^.

Vernalisation represents a critical phase of cold temperature exposure, typically lasting from six to twelve weeks at 0–5 °C, necessary to induce flowering in many plants^31^. In many populations of weeds like *A. myosuroides* and *L. multiflorum*, vernalisation influences their reproductive phenology by hastening flowering. Earlier flowering enables weeds to complete seed production before management interventions, such as herbicide applications^29^. Weeds may undergo vernalisation as either seeds, seedlings, or both, which ensures timely flowering in spring, whether the seedlings emerge in the preceding autumn or shortly before flowering in spring^32^. Certain populations of *A. myosuroides* and *L. multiflorum* exhibit an obligate need for extended cold temperatures to initiate flowering and seed production, known as winter types^31^. However, other populations may undergo behavioural changes to acclimate to the new growing conditions due to factors such as weed control strategies and changes to cropping systems. This adaptation enables them to flower and produce seeds with minimum or no vernalisation requirements. Such populations are termed spring types^31^. Winter types germinate in autumn and are primarily vernalised as seedlings, while spring types undergo vernalisation as seeds during winter, seedlings after germinating in spring, or not at all.

In this study, we investigated variations in germination, flowering, and seed production traits in populations of *A. myosuroides* and *L. multiflorum* in the United Kingdom. Seeds were collected from fields with long-term histories of either autumn or spring cropping systems, referred to herein as parental populations. Our primary objective was to determine if certain UK populations of these species are adapting their germination and growth habits to align with spring cropping practices, building on prior research showing shifts in weed phenology in response to management changes. Specifically, we examined whether populations from spring-cropping fields show enhanced adaptation to vernalisation treatments typical of spring conditions compared to populations from autumn-cropping fields. Spring-like vernalisation conditions are experimental treatments simulating the minimal or absent prolonged cold exposure typical of spring, under which we hypothesise that spring populations will exhibit higher germination rates, earlier flowering, and greater fecundity compared to populations from autumn-cropping backgrounds.

## Materials and methods

### Parental seed collection

**Fig. 1:**
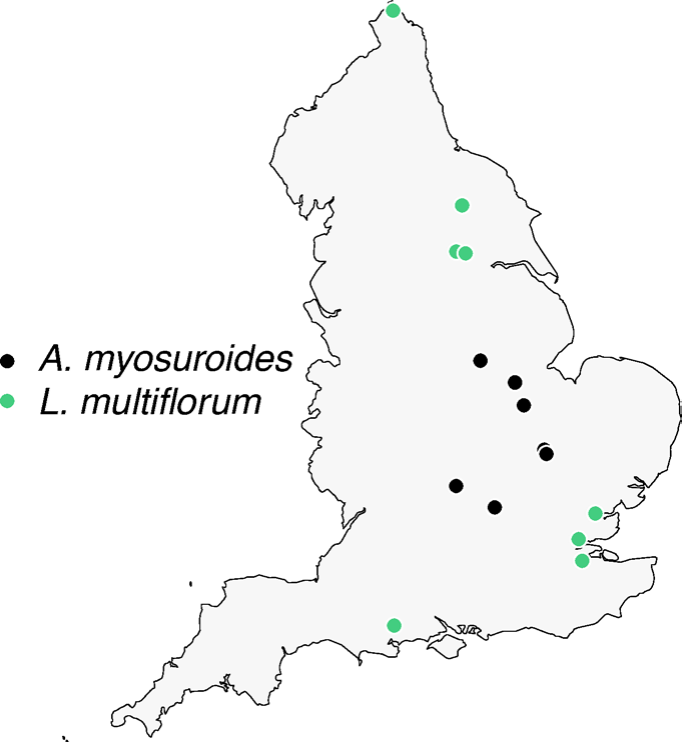
Geographic distribution of *A. myosuroides* and *L. multiflorum* parental populations used in this study, collected from various locations across the UK.

Experiments were conducted between October 2022 and August 2024 to evaluate the adaptation of *Alopecurus myosuroides* and *Lolium multiflorum* based on their germination, flowering, and fecundity characteristics to long-term autumn- and spring-cropping systems. This study was carried out at Niab in Cambridge, United Kingdom. A total of eight *A. myosuroides* and eight *L. multiflorum* parental populations were collected from various UK geographic regions where these species are dominant^33^ (Fig. 1). The selected fields were more than 5 km apart to ensure the independence of the seed samples and minimise the potential for spatial autocorrelation, as reported by Andersson and Espeby^16^.

Seed samples were chosen for comparison between biotypes of *A. myosuroides* and *L. multiflorum* with an autumn or spring cropping history. Suspected spring cropping biotype populations were selected based on high levels of these species in spring crops, coupled with reduced effectiveness of spring cropping as a weed management strategy. Spring- and autumn-based seed samples came from fields that had been under spring or autumn cropping continuously for at least the previous four years. At the time of seed collection, all plants for both species were growing within winter wheat, spring oats, spring barley, or spring corn, spaced across each field. Further information about the fields’ geographic locations and cropping history is shown in Table S1 of the Supplementary Information.

Fully matured *A. myosuroides* and *L. multiflorum* seeds were collected between June and July 2022 and 2021, respectively. Seeds were randomly sampled from at least five plants within each field, maintaining a minimum radius of at least 5 m between collection points. The seeds were then combined to create a composite sample for each field, referred to herein as a ‘population.’ Seeds obtained from autumn- or spring-drilled crops were assumed to have primarily germinated predominantly during the autumn or spring cropping season, respectively. After collection, the seeds were air-dried and stored in paper envelopes under dry laboratory conditions. Before the commencement of each experiment, the seeds were cleaned, and only those with filled caryopses were selected. Filled seeds possess both an embryo and endosperm, indicating their potential for successful germination and growth^34^.

### Germination assessment of parental seed populations

Parental seed of populations from both autumn- and spring-cropping backgrounds underwent germination tests in an incubator with alternating temperature regimes (17 °C light/11 °C dark, 10 h/14 h photoperiod) to assess variability. Warmer temperatures, commonly used for promoting germination in *A. myosuroides* and *L. multiflorum*, were applied to maximise germination success. These seeds were not used for subsequent trait assessments, so vernalisation was not required. Fifty seeds were placed in each Petri dish (90 mm) and covered with aluminium foil to maintain dark conditions, while light treatments remained uncovered throughout the germination period. Each population in each treatment was replicated four times, with 7 ml of distilled water added per Petri dish, and seeds were incubated for five weeks. Germination was assessed at the end of the period by counting germinated and ungerminated seeds.

Although viability tests were not conducted on the ungerminated seeds, we carefully selected seeds with filled caryopses, which are generally indicative of mature and potentially viable seeds^34^. This approach reduced, but did not eliminate, the chance of including non-viable seeds. We, therefore, acknowledge the absence of a viability test as a limitation when interpreting dormancy-related differences between populations.

### Germination assessment of seeds from plants grown under common-garden conditions

We grew autumn and spring parental seeds of *A. myosuroides* and *L. multiflorum* in a glasshouse under standardised common-garden conditions^35^, and the resulting seeds from these plants were collected and are referred to as ‘offspring seed.’ The parental seeds were germinated at 6 °C light/4 °C dark, with an 8 h light/16 h dark cycle in a chilling chamber to induce vernalisation, before being transplanted and grown in an unheated glasshouse. Populations were set 5 m apart, and individual plants were covered with pollination bags during the flowering phase to prevent cross-pollination.

Offspring seeds were subsequently germinated to examine the extent to which maternal growing conditions influenced germination behaviour. Although seeds were collected at physiological maturity during the same period, we did not record environmental data from the source fields during seed development. Thus, we cannot fully account for maternal environmental effects, which may have contributed to the observed trait variability. Germination was assessed following the method described in the previous experiment in an incubator set at 17 °C light/11 °C dark, 10 h/14 h photoperiod.

### Vernalisation treatment to assess flowering and fecundity characteristics

We conducted an experiment between November 2022 and May 2023 to assess flowering and fecundity traits of *A. myosuroides* and *L. multiflorum* parental populations from autumn- and spring-cropping histories using field-collected seed. The plants were subjected to vernalisation treatments at different growth stages. Details of germination and growing conditions are in Table 1. Two-litre pots with a top diameter of 127 mm were filled with commercial sterilized soil and arranged in a warm environment throughout the experiment, with three plants grown in each pot.

**Table 1:** Summary of the germination and growing conditions for the vernalisation treatments.

Abbreviation	Definition	Germination condition	Growth condition
NV	No vernalisation	17 °C light/11 °C dark, 14 h/10 h (germination incubator)	Warm glasshouse (14–26 °C)
GV	Germination vernalisation	6 °C light/4 °C dark, 8 h/16 h (vernalisation room)	Warm glasshouse (14–26 °C)
SV	Seedling vernalisation	17 °C light/11 °C dark, 14 h/10 h (germination incubator)	Vernalisation room (5 °C constant) for 6 weeks followed by transfer to heated glasshouse
GSV	Germination + seedling vernalisation	6 °C light/4 °C dark, 8 h/16 h (vernalisation room)	Vernalisation room (5 °C constant) for 6 weeks followed by transfer to heated glasshouse

Plants were observed daily during the anthesis phase to investigate the flowering time of the two population groups. Flowering was the first appearance of open flowers with visible pollen, indicating active anthesis. The days to first flowering were recorded among the three plants in each replicate. Temperature data were obtained directly from the glasshouse monitoring system, which captured real-time environmental conditions throughout the experimental period. These data were used to estimate the cumulative growing degree days to anthesis. The total number of inflorescences (hereafter referred to as heads) and filled seeds was recorded to assess the fecundity of the populations. The total number of filled seeds was estimated using the average weight of three subsamples for each seed sample.

### Statistical analysis

#### Germination patterns

All statistical analyses were conducted in *R* (v.4.2.3). Germination rates of *A. myosuroides* and *L. multiflorum* populations were analysed using Bayesian generalised linear mixed-effects models (GLMMs) from the *rstanarm* package^36^. This Bayesian approach was selected for its ability to estimate the full posterior distributions of germination probabilities, allowing robust quantification of uncertainty and credible intervals on the random and fixed effects. The model accounted for the effects of cropping background (autumn vs. spring) treatment (light vs. dark) on germination rates, with an interaction term to account for their combined effects. Population was included as a random effect to account for between-population variability. A binomial family was specified to model germination rates as the number of germinated seeds out of the total seeds (germinated + ungerminated). Posterior distributions of germination probabilities were estimated for each background and treatment combination, as well as at the population level. Mean predicted probabilities and 95% credible intervals (CIs) were calculated on the logit scale (but were visualised on the real scale). The model was specified as follows:

1 $$\text{logit}(R)={\beta }_{0}+{\beta }_{1}(\text{Bck})+{\beta }_{2}(\text{Trt})+{\beta }_{3}\left(\text{Bck }:\text{Trt}\right)+{\mu }_{0}\left(\text{Pop}\right)+\varepsilon$$

where *R* is the germination probability, *β*_0_ is the intercept, *β*_1_, *β*_2_, and *β*_3_ are the fixed-effect coefficients for background (Bck), treatment (Trt), and their interaction, respectively, ${\mu }_{0}$ represents random effects for population (Pop), and *ε* is the residual error.

#### Flowering time

Flowering time was analysed using cumulative growing degree days (GDD) to first flowering data collected from *A. myosuroides* and *L. multiflorum* populations vernalised at different growth stages. A linear model was applied to test for differences in GDD between treatments and cropping backgrounds, using the interaction term between these factors to account for dependencies. We chose a linear model because GDD is a continuous variable that approximates normality, making it well-suited to linear modelling while providing a straightforward and computationally efficient framework. The model is specified as follows:

2 $$GDD={\beta }_{0}+{\beta }_{1}(\text{Bck})+{\beta }_{2}(\text{Trt})+{\beta }_{3}\left(\text{Bck }:\text{Trt}\right)+\varepsilon$$

where *β₀* is the intercept, *β*_1_, *β*_2_, and *β*_3_ are coefficients for background (Bck), treatment (Trt), and their interaction, respectively. The population-level effect had no impact on flowering time and was excluded from the final model. Mean GDD values were aggregated by population background and treatment. Density plots were generated using the *ggplot2* package to visualise the mean GDD distributions for both species under various vernalisation treatments.

#### Fecundity traits

To evaluate fecundity traits, heads and seeds per plant, we used Poisson generalized linear mixed-effects models (GLMMs) from the *lme4* package^37^. A Poisson distribution was appropriate given the count-based nature of the response variables, while observation-level random effects (OLRE, explained by Harrison (2014)) were included to address any over-dispersion in the models^38^. The marginal and conditional R^2^ for the final models were calculated using the *MuMIN* package to assess model fit^38^. The results were back-transformed by exponentiating the model’s log-transformed estimates to the original scale (i.e., count scale) for interpretation. The model was specified as:

3 $$R={\beta }_{0}+{\beta }_{1}(\text{Bck})+{\beta }_{2}(\text{Trt})+{\beta }_{3}\left(\text{Bck }:\text{Trt}\right)+{\mu }_{0}\left(\text{Pop}\right)+\varepsilon$$

where *R* represents the response variable (seed or head), *β₀* is the intercept, *β*_1_, *β*_2_, and *β*_3_ represent the fixed-effect coefficients for background (Bck), treatment (Trt), and their interaction, respectively, *μ*_0_(Pop) represents the random effects for population, and *ε* is the error term.

## Results

### Germination characteristics of populations from autumn- and spring-cropping backgrounds

**Fig. 2:**
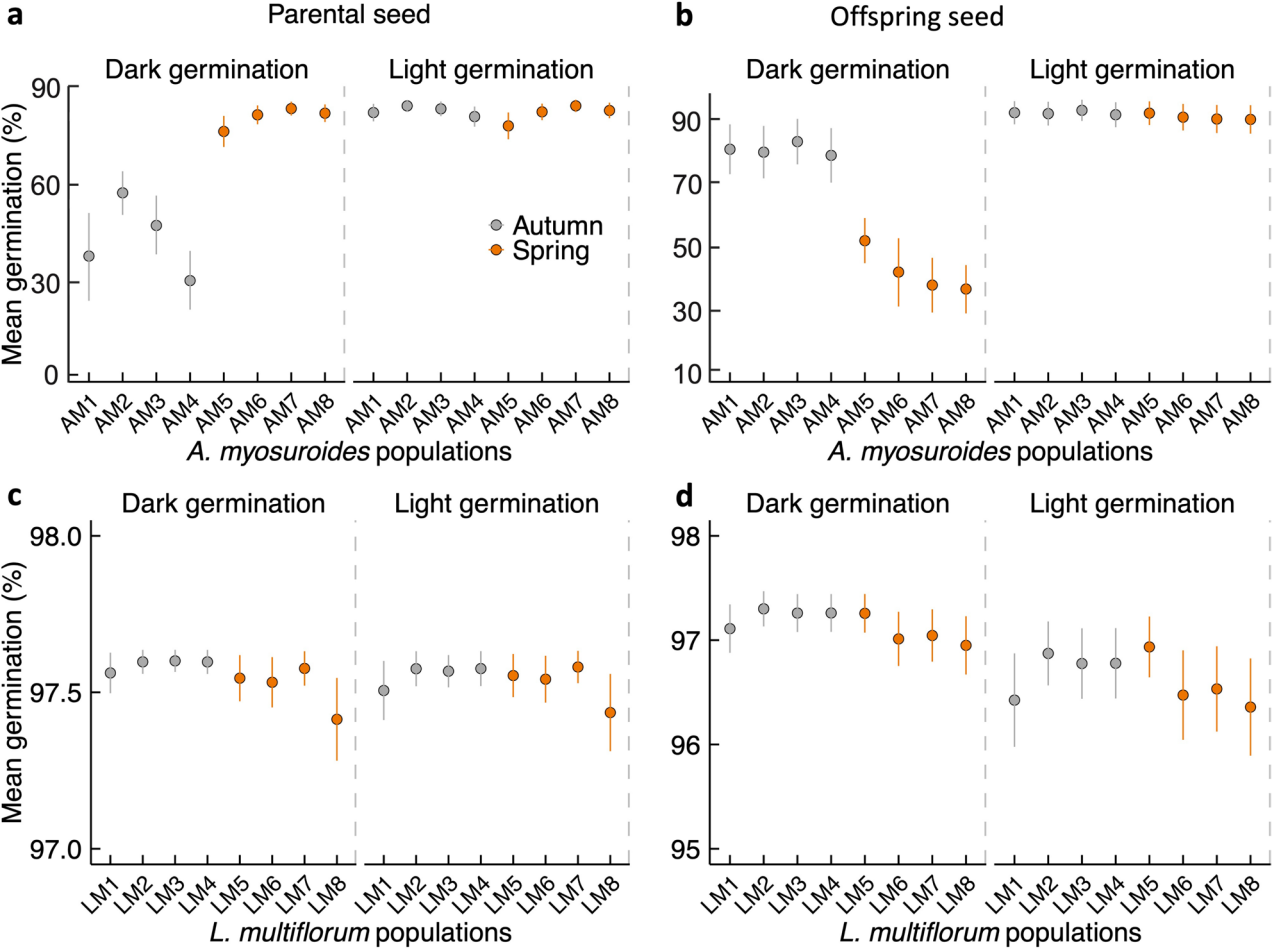
Variation in germination patterns of *Alopecurus myosuroides* (**a**, **b**) and *Lolium multiflorum* (**c**, **d**) in response to light and darkness across parental and offspring seeds from autumn- and spring-cropping backgrounds. Vertical bars represent the mean predicted germination rates, with error bars indicating the 95% credible intervals around the predicted population means.

Germination rates, logit-transformed and converted to percentages using the logistic transformation, are reported with 95% credible intervals (CIs). Under dark treatment conditions, parental seeds of *Alopecurus myosuroides* from a spring-cropping background exhibited higher germination rates than those from an autumn-cropping background (Fig. 2a; Table 2). Notably, the offspring seeds of *A. myosuroides* from an autumn-cropping background demonstrated a substantial increase in germination rates under dark conditions compared to seeds from a spring-cropping background (Fig. 2b; Table 2). Offspring seeds of *A. myosuroides* from both autumn- and spring-cropping histories maintained higher germination rates under light conditions (90% and 89.7%, respectively) (Fig. 2b). In addition, parental and offspring seeds of *Lolium multiflorum* consistently exhibited high germination rates regardless of light exposure (Fig. 2c,d), and were therefore not included in Table 2.

**Table 2:** Germination rates (%, with 95% credible intervals) for parental and offspring seeds of *A. myosuroides* under dark conditions, comparing populations from autumn- and spring-cropping backgrounds.

Species	Seed type	Light condition	Cropping background	Germination (%)	95% CI
*A. myosuroides*	Parental	Dark	Autumn	45.7	26.0–61.4
			Spring	84.5	70.5–88.7
*A. myosuroides*	Offspring	Dark	Autumn	80.7	70.2–90.0
			Spring	41.6	30.3–54.8

Only significant comparisons are shown. *L. multiflorum* is excluded due to uniformly high germination across treatments.

### Differential growing degree day requirements to first flowering

**Fig. 3:**
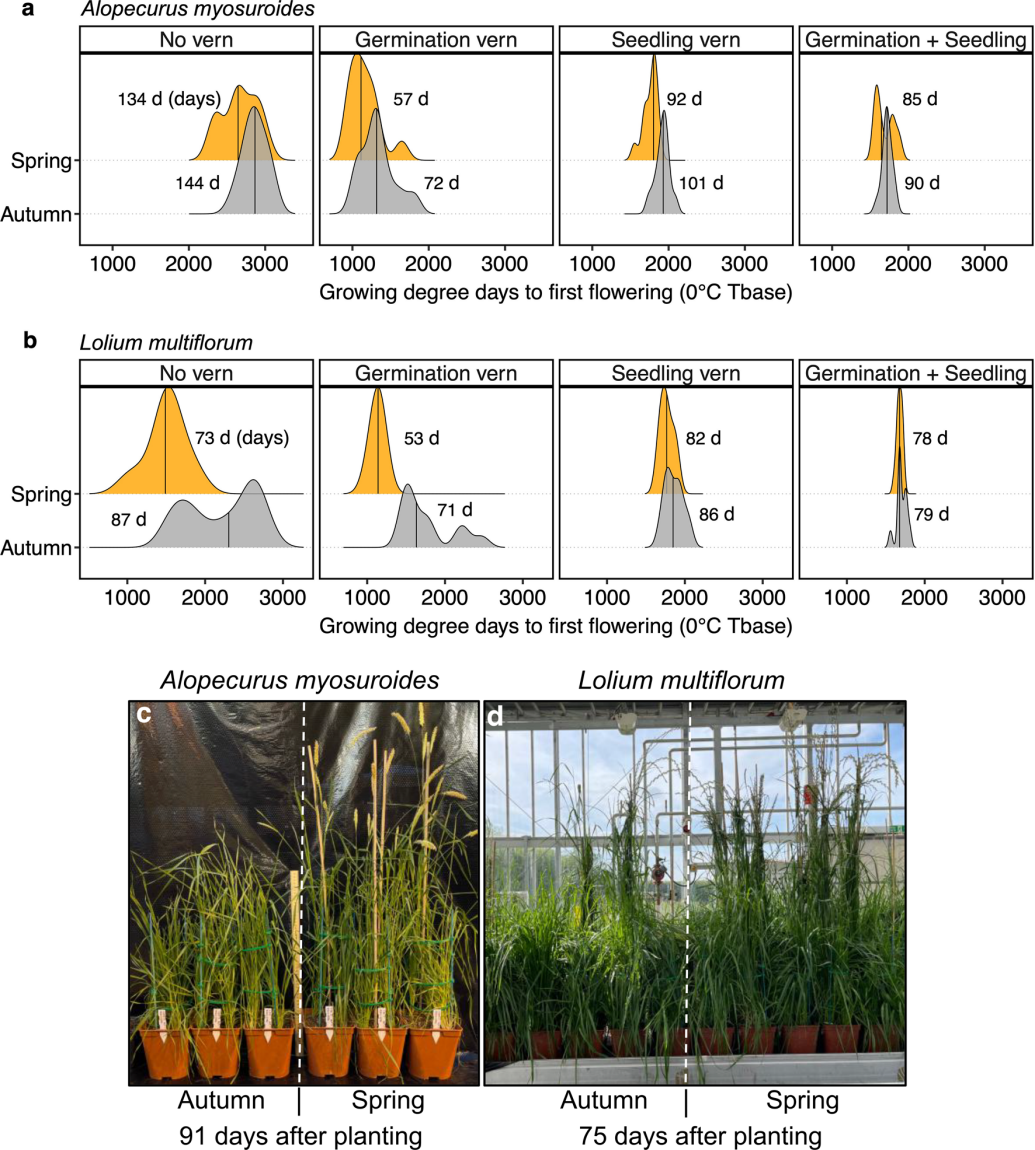
Comparison of growing degree days (GDD) for parental populations of *A. myosuroides* (**a**) and *L. multiflorum* (**b**) from autumn- and spring-cropping backgrounds under different vernalisation treatments. The vernalisation treatments are No vern, no vernalisation; Germination vern, germination vernalisation; Seedling vern, seedling vernalisation; Germination + Seedling, germination and seedling vernalisation. The vertical solid lines represent the mean thermal time **(**GDD) from planting to flowering of populations from the same background within each treatment. The width of each curve shows the overall flowering window under each treatment. The average number of days (d) to first flowering, corresponding to the GDD values on the x-axis, is annotated within each subplot. Visual comparison of distinct flowering stages observed in autumn and spring populations of *A. myosuroides* and *L. multiflorum* under no vernalisation and germination vernalisation treatment, respectively (**c**, **d**).

Populations of both species from spring-cropping backgrounds exhibited earlier flowering under vernalisation treatments aligned with their parental cropping history. Specifically, *A. myosuroides* populations from spring backgrounds flowered earlier than those from autumn backgrounds under both no vernalisation (NV) and germination vernalisation (GV) treatments (Fig. 3a; Table 3). Similarly, *L. multiflorum* populations from spring backgrounds also flowered earlier under NV and GV treatments (Fig. 3b; Table 3). In NV treatments, populations from a spring-cropping history flowered 10 days earlier than those from an autumn-cropping background in *A. myosuroides* and 14 days earlier in *L. multiflorum*. Under GV, the differences were more pronounced, with populations from a spring-cropping background flowering 15 days earlier than autumn populations in *A. myosuroides* and 18 days earlier in *L. multiflorum*. Although trends toward earlier flowering were observed for spring-cropping populations under SV treatments in both species and GSV treatments in *A. myosuroides*, the differences were not statistically significant. Variations among individual populations are shown in Fig. S1 of the Supplementary Information**.**

**Table 3:** Flowering time (growing degree days (GDD) and calendar days) to first flowering for parental seeds of *A. myosuroides* and *L. multiflorum* under no vernalisation (NV) and germination vernalisation (GV) treatments.

Species	Vernalisation treatment	Cropping background	GDD to 1st flowering	Days to 1st flowering	*P*-value
*A. myosuroides*	NV	Autumn	2864	134	< 0.05
		Spring	2666	144	
	GV	Autumn	1331	72	< 0.05
		Spring	1158	57	
*L. multiflorum*	NV	Autumn	2212	87	< 0.0001
		Spring	1504	73	
	GV	Autumn	1771	71	< 0.0001
		Spring	1130	53	

Only significant differences between cropping backgrounds are shown. GDD refers to the cumulative thermal time from planting to first flowering.

### Number of heads per plant

**Fig. 4:**
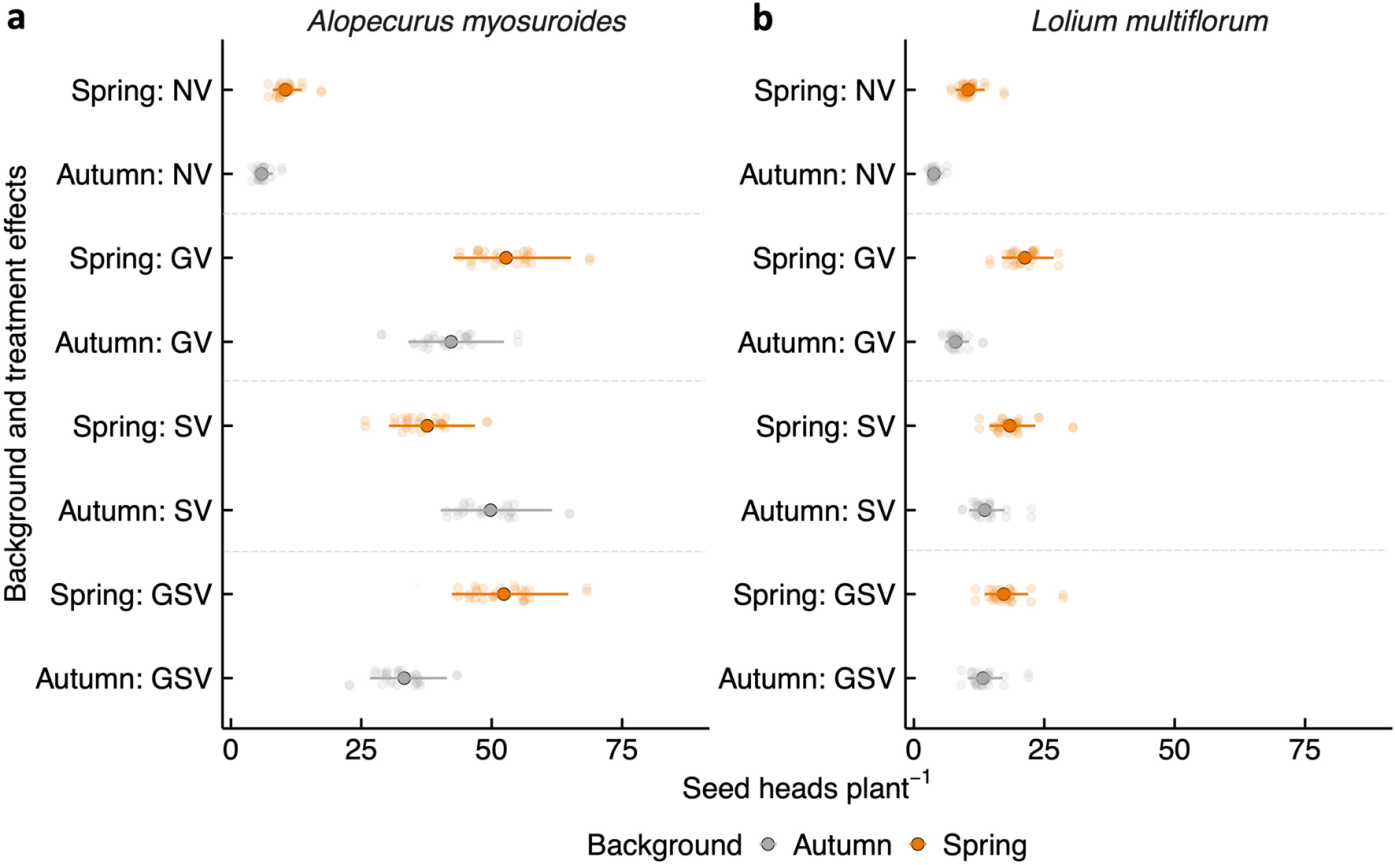
The interaction plots for the mean number of heads per plant, estimated on a log scale, for the autumn and spring parental populations of *A. myosuroides* (**a**) and *L. multiflorum* (**b**) across vernalisation treatments. The treatments are NV (no vernalisation), GV (germination vernalisation), SV (seedling vernalisation), and GSV (germination and seedling vernalisation). The dots represent the population-level means for the estimated number of heads per plant, and error bars represent 95% confidence intervals of the mean.

In *Alopecurus myosuroides*, the number of heads per plant was significantly higher in populations from a spring-cropping background compared to those from an autumn-cropping background under vernalisation treatments typical of spring-cropping conditions. Specifically, under no vernalisation, spring-cropping populations produced more heads than autumn-cropping populations (spring mean: 11 vs. autumn mean: 6, *P* < 0.05). Similarly, under germination and seedling vernalisation treatments, spring-cropping populations had significantly more heads than autumn-cropping populations (spring mean: 52 vs. autumn mean: 33, *P* < 0.05; Fig. 4a). For *Lolium multiflorum*, plants from spring-cropping populations produced significantly more heads per plant under no vernalisation (spring mean: 10 vs. autumn mean: 4, *P* < 0.01) and germination vernalisation treatments (spring mean: 21 vs. autumn mean: 8, *P* < 0.001) than populations from an autumn-cropping background (Fig. 4b). It is noteworthy that not all plants in the no-vernalisation treatment produced heads, which likely contributed to the lower overall means observed in this treatment compared to the vernalisation treatments. Variability was observed between populations from the same cropping background and treatment across both species.

### Total viable seed production

**Fig. 5:**
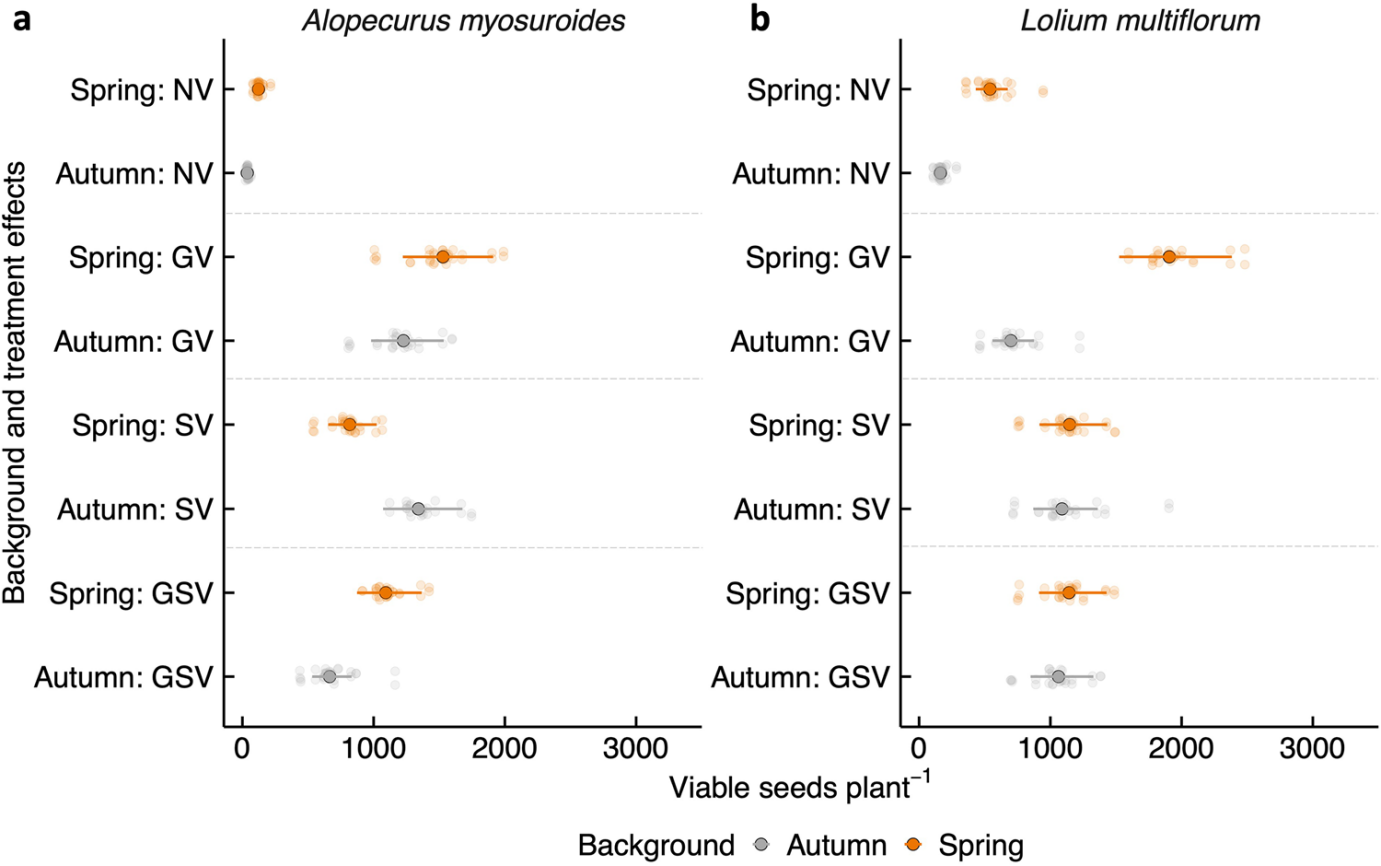
Interaction plots showing the mean number of seeds per plant, estimated on a log scale, for the autumn and spring parental populations of *A. myosuroides* (**a**) and *L. multiflorum* (**b**) under different vernalisation treatments. The treatments include NV (no vernalisation), GV (germination vernalisation), SV (seedling vernalisation), and GSV (germination and seedling vernalisation). Dots indicate population-level means for the estimated number of seeds per plant, while error bars represent 95% confidence intervals of the means.

The comparative patterns between spring and autumn populations were consistent in the number of heads (Fig. 4), and the number of viable seeds (Fig. 5). In *A. myosuroides*, populations from a spring-cropping background produced significantly more seeds under no vernalisation (spring mean: 123 vs. autumn mean: 37, *P* < 0.001) and under germination plus seedling vernalisation treatments (spring mean: 1092 vs. autumn mean: 664, *P* < 0.01; Fig. 5a). Conversely, under seedling vernalisation, plants from an autumn-cropping background produced significantly more seeds than those from a spring-cropping background (autumn mean: 1340 vs. spring mean: 817, *P* < 0.05). For *L. multiflorum*, populations from a spring-cropping background also produced significantly more seeds per plant than autumn-cropping populations under no vernalisation (spring mean: 541 vs. autumn mean: 162, *P* < 0.001). Similarly, under germination vernalisation, spring-cropping populations produced more than double the seeds compared to autumn populations (spring mean: 1906 vs. autumn mean: 699, *P* < 0.001; Fig. 5b). However, variations were observed between populations within the same cropping background and treatment.

## Discussion

Populations of *A. myosuroides* and *L. multiflorum* exhibited phenotypic evidence of potential adaptation to spring cropping systems, including increased germination rates, earlier flowering, and greater fecundity under spring-like vernalisation conditions.

The higher germination rates of spring parental populations under dark conditions than autumn populations likely reflect the selection pressure from the spring cropping system. In spring-cropping environments, seeds are exposed to conditions favouring traits such as reduced light requirements, enabling germination under competitive conditions with limited light. Over time, these populations may have evolved lower dormancy thresholds and reduced dependency on light as a germination cue. Additionally, *A. myosuroides* populations from spring cropping systems may have adopted bet-hedging and risk-spreading strategies. These strategies promote germination under suboptimal cues, such as darkness, ensuring that some seeds germinate regardless of environmental conditions, thereby enhancing survival and reproductive success^22^.

Seed ripening conditions may further contribute to these differences. Seeds from a spring-cropping history ripen under longer daylight and warmer temperatures, potentially reducing their light requirements for germination. Conversely, seeds from autumn systems, maturing under cooler and shorter-day conditions, may exhibit stronger reliance on light as a germination trigger^39^. Similar patterns have been observed in *A. myosuroides* and *Bidens pilosa*, where substantial germination occurs in spring, with many seeds germinating in darkness^21,40^.

However, the observed reversal in *A. myosuroides* germination trends in seed produced under a common-garden setup, where autumn populations germinated more readily in darkness than spring populations, cannot be solely attributed to maternal environmental effects, although these likely contribute. Such effects, where the environment experienced by the parent plant during seed development influences offspring traits, may have played a role^22,41^. While the common-garden conditions aimed to standardize plant growth and seed maturation environment, source field conditions (cooler and shorter days for autumn-cropping plants versus warmer and longer days for spring-cropping plants) may have shaped inherent differences between populations^42^. Other field factors, including crop canopy structure and resource availability, might also have influenced dormancy traits. However, the uniform growth conditions likely minimized the immediate maternal effects, suggesting that the reversal reflects an interaction between genetic and environmental influences^43^. In natural conditions, parent plants may “prime” seeds for specific germination responses, while controlled environments can reveal underlying genetic predispositions^44^. This highlights the complex interplay between environmental cues and heritable traits in shaping germination behaviour.

The low dormancy observed across seeds from both cropping histories may reflect seed production under uniform conditions, ambient storage promoting after-ripening, and germination tests conducted under optimal conditions. These factors likely masked dormancy expression, though reduced dormancy may confer a selective advantage under spring-cropping systems, where rapid and synchronous emergence is beneficial.

In terms of flowering phenology, spring populations of both *A. myosuroides* and *L. multiflorum* exhibited earlier flowering across spring-like vernalisation treatments, including no vernalisation, compared to autumn populations. However, considering these species typically require chilling exposure to flower^45,46^, this reflects an evolutionary shift towards reduced vernalisation requirements in response to cropping system pressures. Similar trends have been documented in *Brassica* species, where rising spring temperatures drive earlier flowering^16,47,48^. Unlike autumn-germinating plants, spring-germinating plants encounter favourable climatic conditions immediately after emergence, enabling rapid growth and development. This accelerated life cycle allows them to complete seed production within a shorter timeframe, providing a competitive advantage by avoiding summer stresses such as drought or competition from late-germinating weeds^49^. Interestingly, *L. multiflorum* populations from autumn-cropping systems displayed a bimodal flowering pattern, suggesting staggered flowering timing within the same population group. Such plasticity in flowering may serve as a risk-spreading mechanism in response to unpredictable environmental conditions, increasing population persistence across a broader range of field conditions^50–52^.

The higher appearance rate of *A. myosuroides* leaves during early spring germination facilitates faster development and earlier seed production in spring cereals^53^. Furthermore, autumn and spring cohorts may also result from the selection of two different biotypes with distinct germination and flowering requirements^45^. These differences likely reflect adaptive strategies to seasonal cues: spring-cropping populations, with lower vernalisation requirements, initiate reproduction more readily under limited cold exposure, enabling higher seed production despite a shorter growing season. In contrast, autumn populations require prolonged vernalisation, particularly as seedlings, and thus perform better when extended cold exposure is induced, aligning with their overwintering life cycle.

Fecundity differences between *A. myosuroides* and *L. multiflorum* populations further underscore their adaptive potential. Spring populations of both species showed increased seed production under no vernalisation and minimal vernalisation treatments, highlighting their ability to thrive despite reduced chilling exposure. Although no vernalisation treatments had lower fecundity than other treatments, these results suggest that vernalisation is not an absolute requirement in spring types of *A. myosuroides* and *L. multiflorum*. The variability observed among the two biotypes may indicate the role of genetic diversity and bet-hedging strategies in the reproductive phenology of these grasses^50,54^. In the case of *A. myosuroides* and *L. multiflorum*, the production of both spring and winter biotypes within populations may reflect an adaptive mechanism to persist despite variable vernalisation opportunities, weed control practices, and climate conditions^55,56^. Individuals from spring-cropping history undergo vernalisation as seeds and during the seedling stage, or they may experience minimal or no vernalisation^32^. Fecundity is a fundamental aspect of fitness for weediness in an agricultural setting. If changing climates select for even higher seed production, these species will become even more difficult to control^13,57^. Meanwhile, crops, such as winter cereals, show similar trends in flowering time, head emergence, and seed production under warmer spring temperatures, meaning their critical growing time will continue to overlap with the weeds^3,4,13^. While late-germinating weeds may have a reduced impact on crop yields, they will still produce seeds and substantially contribute to the seedbank for future seasons^26^.

In summary, the interaction between genetic and environmental effects should not be overlooked. Plants may change their traits during evolution due to epigenetic modifications or transient abiotic environmental changes^12,58^. Some of these traits, known as labile traits, may persist only when plants are exposed to specific environmental conditions, while others can be passed on to the offsprings^59^. Populations of *A. myosuroides* exhibit heritability of phenotypic variation in flowering traits such as time to flower and flower head number in response to non-chemical control methods^60^. Such methods include removing weed heads prior to harvest, but the efficacy of this technique is dependent on the height differential between crop and weed. This interplay underscores the need for further investigation into the heritability and genetic basis of adaptation in *A. myosuroides* and *L. multiflorum*. Future studies could employ genome-wide association approaches or epigenetic profiling to identify specific loci or regulatory mechanisms underlying these adaptive traits, particularly in response to cropping practices such as delayed drilling. By exploring these mechanisms, we can better predict their responses to changing agricultural practices and develop more effective weed management strategies.

## Conclusions

The adaptive capability of weeds plays a crucial role in their ability to withstand diverse selection pressures, including human-imposed control measures and changing environmental conditions. Focusing on germination, flowering, and fecundity traits, we demonstrated some evidence of local adaptation in populations of *A. myosuroides* and *L. multiflorum* in the UK, particularly in flowering and fecundity responses under conditions consistent with the spring cropping systems. In many instances, seeds from a spring-cropping background showed increased performance compared to those from an autumn-cropping system under spring-like conditions. While germination patterns showed some differentiation, the most consistent evidence of adaptation came from later life-history traits. Our study provides crucial phenotypic evidence of adaptation and lays the groundwork for further mechanistic exploration. While spring cropping can help mitigate the impact of *A. myosuroides* and *L. multiflorum*, weeds adapt over time. We, therefore, recommend integrating seasonal rotations within crop establishment windows as a proactive strategy to address weed adaptation and promote sustainable weed management.

## Electronic supplementary material

Below is the link to the electronic supplementary material.

Supplementary Material 1

## Data Availability

Data that support the findings of this study are available from the corresponding author upon request.
