# Correction: Adaptation of grassweeds to spring cropping through changes in germination, flowering time and fecundity

**DOI:** 10.1038/s41598-025-16466-8

**Published:** 2025-09-11

**Authors:** Jasper Kanomanyanga, John Cussans, Stephen Moss, Erick Ober, Chun Liu, Shaun Coutts

**Affiliations:** 1 Lincoln Institute for Agri-Food Technology, University of Lincoln, Lincoln, UK; 2 Niab, Cambridge, UK; 3 ADAS Boxworth, Cambridge, UK; 4 Stephen Moss Consulting, Harpenden, Hertfordshire, UK; 5 Syngenta, Jealott’s Hill International Research Centre, Bracknell, Berkshire, UK

Correction to: *Scientific Reports* 10.1038/s41598-025-04664-3, published online 01 July 2025

In the original version of this Article, panels c and d were omitted from Figure 3.

The original Article has been corrected.

