# Supplementary material for: Adaptation of grassweeds to spring cropping through changes in germination, flowering time and fecundity
Source: Sci Rep. 2025 Jul 1;15:21492. doi: 10.1038/s41598-025-04664-3 (PMC12215899; doi:10.1038/s41598-025-04664-3)
Supplement: Supplementary file 1 — Supplementary Material 1 [file 41598_2025_4664_MOESM1_ESM.docx]

**Adaptation of grassweeds to spring cropping through changes in germination, flowering time and fecundity**

Jasper Kanomanyanga^1,2^, John Cussans^2,3^, Stephen Moss^4^, Erick Ober^2^, Chun Liu^5^, Shaun Coutts^1,*^

^1^Lincoln Institute for Agri-food Technology, University of Lincoln, Lincoln, UK ([jasper.kanomanyanga@niab.com](mailto:jasper.kanomanyanga@niab.com); [scoutts@lincoln.ac.uk](mailto:scoutts@lincoln.ac.uk))

^2^Niab, Cambridge, UK ([eric.ober@niab.com](mailto:eric.ober@niab.com))

^3^ADAS Boxworth, Cambridge, UK ([john.cussans@adas.co.uk](mailto:john.cussans@adas.co.uk))

^4^Stephen Moss Consulting, Harpenden, Hertfordshire, UK ([alopecurus@aol.com](mailto:alopecurus@aol.com))

^5^Syngenta, Jealott's Hill International Research Centre, Bracknell, Berkshire, UK ([chun.liu@syngenta.com](mailto:chun.liu@syngenta.com)).

**Corresponding author**

Shaun Coutts ([scoutts@lincoln.ac.uk](mailto:scoutts@lincoln.ac.uk))

**Supplementary information**

**Table S1 |** Geographical locations and cropping histories of *Alopecurus myosuroides* and *Lolium multiflorum* populations collected from across the United Kingdom.

| **Species** | **Location (UK)** | **Population** | **Year of collection** | **Cropping history until collection year** | **Collected from** | **Background** |
| --- | --- | --- | --- | --- | --- | --- |
| *Alopecurus myosuroides* | Childerley, Cambridgeshire | AM1 | 2022 | Repeated winter cropping since 1960 | Winter wheat | Autumn |
|  | Hardwick, Cambridgeshire | AM2 | 2022 | Repeated winter copping for >10 years | Winter wheat | Autumn |
|  | Elton, Nottinghamshire | AM3 | 2022 | Four years of repeated winter cropping | Winter wheat | Autumn |
|  | Bourne, Lincolnshire | AM4 | 2022 | > 5 years of repeated winter cropping | Winter wheat | Autumn |
|  | Tring, Buckinghamshire | AM5 | 2022 | Six years of repeated spring barley | Winter wheat | Spring |
|  | Tring, Buckinghamshire | AM6 | 2022 | Five years of repeated spring barley cropping | Winter wheat | Spring |
|  | Somerton, Oxfordshire | AM7 | 2022 | Four years of repeated spring barley | Spring oats | Spring |
|  | Marholm, Cambridgeshire | AM8 | 2022 | > 4 years of repeated spring cropping | Spring barley | Spring |
| *Lolium multiflorum* | Berwick, Northumberland | LM1 | 2021 | > 5 years of repeated winter cropping | Winter wheat | Autumn |
|  | Redcar, Northeast Yorkshire | LM2 | 2021 | > 5 years of repeated winter cropping | Winter wheat | Autumn |
|  | Rochester, Kent | LM3 | 2021 | > 5 years of repeated winter cropping | Winter wheat | Autumn |
|  | Horndon-on-the-Hill, Essex | LM4 | 2021 | > 5 years of repeated winter cropping | Winter wheat | Autumn |
|  | Biggin, North Yorkshire | LM5 | 2021 | > 5 years of repeated spring cropping | Spring barley | Spring |
|  | Maldon, Essex | LM6 | 2021 | > 5 years of repeated spring cropping | Spring corn | Spring |
|  | Selby, North Yorkshire | LM7 | 2021 | > 5 years of repeated spring cropping | Spring corn | Spring |
|  | Wimbourne, Dorset | LM8 | 2021 | > 5 years of repeated spring cropping | Spring barley | Spring |

**Supplementary Figures**

**Fig. S1** **|** Growing degree days (GDD) to first flowering for the parental populations of *A. myosuroides* (**a**) and *L. multiflorum* (**b**) from autumn- and spring-cropping histories under different vernalisation treatments. The vernalisation treatments are NV, no vernalisation; GV, germination vernalisation; SV, seedling vernalisation; GSV, germination and seedling vernalisation. The vertical solid lines represent the mean cumulative GDD of a population, whereas the width of each curve represents the overall flowering window of a population in each treatment.
